# Longitudinal effects of parental, child and neighborhood factors on moderate-vigorous physical activity and sedentary time in Latino children

**DOI:** 10.1186/s12966-014-0108-x

**Published:** 2014-09-04

**Authors:** Nancy F Butte, Steven E Gregorich, Jeanne M Tschann, Carlos Penilla, Lauri A Pasch, Cynthia L De Groat, Elena Flores, Julianna Deardorff, Louise C Greenspan, Suzanna M Martinez

**Affiliations:** Department of Pediatrics,Baylor College of Medicine, USDA/ARS Children’s Nutrition Research Center, 1100 Bates Street, Houston, TX 77030-2600 USA; Division of General Internal Medicine, University of California at San Francisco, 3333 California St. Ste. 335, San Francisco, CA 94118 USA; Department of Psychiatry, University of California at San Francisco, Box 0848, San Francisco, CA 94143-0848 USA; Counseling Psychology Department, School of Education, University of San Francisco, 2130 Fulton Street, San Francisco, CA 94118 USA; Division of Community Health and Human Development, School of Public Health, 50 University Hall, University of California at Berkeley, Berkeley, CA 94720-736 USA; Kaiser Permanente San Francisco, Department of Pediatric, 2200 O’Farrell Street, San Francisco, CA 94115 USA; Division of General Pediatrics, University of California at San Francisco, San Francisco, CA 94143-0848 USA

**Keywords:** Physical activity patterns, Accelerometers, Childhood obesity, Maternal factors, Paternal factors, Education level, Acculturation, Household income, Household size, Environment, Disorder, Victimization

## Abstract

**Background:**

Moderate-vigorous physical activity (%MVPA) confers beneficial effects on child musculoskeletal health, cardiovascular fitness, and psychosocial well-being; in contrast, sedentary time (%SED) is emerging as a risk factor for health. This study aimed to identify parental, child and neighborhood factors influencing longitudinal assessments of body mass index (BMI) and activity patterns among Latino children, and to estimate lagged and cross-lagged effects between child BMI, %MVPA and %SED.

**Methods:**

A longitudinal design with assessments at baseline, 1 and 2 years follow-up (FU) was used to evaluate the effects of maternal and paternal factors (BMI, age, education level, acculturation, household income and household size), child factors (gender, age, BMI, pubertal status) and neighborhood factors (disorder, victimization) on child BMI, %MVPA and %SED, expressed as a percent of awake time, in 282 Latino children ages 8–10 y and their parents. This study was restricted to families with a mother and biological father or father figure in the child’s life.

**Results:**

Across time, total daily accelerometer counts (p = 0.04) and steps decreased (p = 0.0001), %SED increased (p = 0.0001), and %MVPA decreased (p = 0.02). Moderate lagged effects or tracking was seen for %MVPA and %SED (p = 0.001). %MVPA varied by gender (5.5% higher in boys than girls, p = 0.0001); child age (−0.4% per year, p = 0.03), and child BMI in boys only (−0.22%, p = 0.0002). Negative effects of paternal age, maternal education and maternal changes in BMI on %MVPA also were seen. %SED increased with child age (2.5% higher per year, p = 0.0001). Positive effects of paternal acculturation, maternal change in BMI, paternal age, and negative effects of household size on %SED were observed. A cross-lagged positive effect of BMI at FU1 on %SED at FU2 was observed for boys and girls (p = 0.03). Neighborhood disorder and victimization were not significant predictors of child BMI, %MVPA or %SED.

**Conclusion:**

The major child determinants of physical activity (age, gender and BMI) and minor parental influences (maternal BMI and education, paternal age and acculturation) should be considered in designing interventions to promote %MVPA and reduce %SED among Latino children as they approach adolescence.

## Background

Regular physical activity is considered essential for the health and development of children. Positive effects of physical activity on musculoskeletal health, cardiovascular fitness, and psychosocial well-being are well recognized, as is the lack of physical activity on excess adiposity, elevated blood lipids, hypertension and glucose intolerance [1,2]. Based on prevailing evidence, the US Department of Health and Human Services (DHHS) recommends ≥60 min/d of moderate-vigorous physical activity (MVPA) for children [3]. Sedentary behaviors have emerged more recently as a health risk with consequences independent of the presence or absence of physical activity [4]. Sedentary behaviors are classified as low energy expenditure while sitting or reclining, and in children encompass activities such as schoolwork, motorized transportation, television viewing, playing video games and using the computer, and have been associated with increased adiposity, adverse cardiometabolic and diabetes risk profiles [5]. DHHS recommends that children aged 2 and older should spend no more than 2 h/d watching television or using a computer (except for school work) [3].

In the US, childhood obesity afflicts a disproportionate number of Latino children [6], and yet the contribution of physical activity and sedentary behaviors to the development of obesity is unclear in this high risk population. Many Latino children are from low socioeconomic status (SES) families living in urban environments with high crime, neighborhood disorder, and limited access to parks and recreational facilities – circumstances that are not conducive to physical activity [7]. Physical activity patterns in Latino children have been assessed objectively using activity devices in a number of studies, but the results and their implications are mixed. In the 2003–2004 NHANES, accelerometers were used to measure physical activity in a nationally representative sample of white, Mexican American and African American children. Mexican American children had similar or higher levels of MVPA than white and African American children [8]. Based on a secondary analysis on the NHANES data, Mexican American children from low-, middle- and high-income households were found to be equally or more active than their white and African American counterparts [9]. In Houston, Texas, 62% of Latino children, ages 4 to 19 y, from low SES families spent ≥60 min/d in MVPA; however, the percent of children meeting the recommendation decreased markedly with age [10]. More recently, MVPA was measured in 483 Latino and African American children, ages 9–12 y, residing in Houston; only 23% of the children met the MVPA recommendation [11]. In inner-city Philadelphia, Latino children had lower levels of MVPA than the other racial/ethnic groups; only 19% met the recommendation of daily MVPA [12]. Discrepant findings between studies, in part, are attributable to differences in study design, population sampling, accelerometer manufacturer and thresholds used to define MVPA, but also to differences in regional sociodemographic factors influencing physical activity patterns in children. Consistently across all these studies, age and gender were salient factors: Latino boys exhibited significantly more MVPA than girls, and MVPA decreased linearly with age. Little is known regarding other potentially important child and sociodemographic factors. Furthermore, the extant cross-sectional studies can only elucidate associations, not inform causality between physical activity patterns and child outcomes. Therefore, longitudinal studies using objective measures of activity are needed to determine the temporal patterns and determinants of physical activity sedentary time (SED) in Latino children.

The specific objectives of this study were 1) to identify parental, child and neighborhood factors influencing longitudinal assessments of child BMI, %MVPA and %SED, expressed as a percent of awake time, over a two-year period among 282 Latino children, aged 8 to 10 years at baseline; 2) to estimate the lagged effects of child BMI, %MVPA and %SED over a two-year period; and 3) to test for cross-lagged effects between child BMI, MVPA and %SED over a two-year period.

## Methods

### Study design

A longitudinal design was used to identify parental, child and environmental factors influencing assessments of child BMI, %MVPA and %SED among Latino children, ages 8 to 10 years at baseline, living in the San Francisco Bay Area. The study design called for repeated assessments at baseline, 1-year (FU1), and 2-year follow-ups (FU2) for children as well as their parents. Families were eligible if the mother self-identified as being of Mexican origin (whether born in the US or Mexico), a child living in the mother’s household was 8–10 years of age, and the child had no major illnesses. Families were eligible whether or not fathers participated, but every effort was made to recruit fathers. If the father did not reside in the same household as the mother and child, the biological father living apart or a primary father figure was identified (i.e., residential parental figure) and recruited to participate. To consider the effects of both mothers’ and fathers’ factors on their children’s BMI and physical activity, the analyses reported here were restricted to families with a mother and biological father or father figure in the child’s life. The study was approved by the University of California at San Francisco, Baylor College of Medicine and Kaiser Permanente institutional review boards.

Families were recruited between 2007–2009 from the membership lists of Kaiser Permanente Northern California, an integrated health care delivery organization. Parents were sent letters introducing the research, were telephoned, screened for eligibility, and invited to participate in the study. Bilingual interviewers obtained parental informed consent and child assent to participate in the research.

At each time point, separate in-home interviews of the child, the mother, and the father (or father figure) were conducted by trained bilingual/bicultural research assistants, in each participant’s preferred language. Interviewers recorded responses to the questionnaires in laptop computers and measured family members’ height and weight. A 3-day assessment of the child’s physical activity was ascertained by accelerometry.

### Measures

#### Sociodemographics

Demographic variables included child gender and age, and parents’ years of education, income, occupational status, number of household occupants, and acculturation reported at baseline. To determine household income, mothers were asked to estimate annual pretax incomes of all individuals living in the home. Occupational status [13] ranged from lowest (=1, unskilled worker) to highest (=9, major professional). Acculturation was assessed using the Spanish and English Language Use subscales of the Bidimensional Acculturation Scale for Latinos [14]. Each subscale consisted of five items, scored from never (=1) to always (=5) (alphas = 0.88–0.94).

Parental perceptions of neighborhood context/risk/crime were assessed using the Disorder and Victimization subscales of the Neighborhood Context Scale [15]. The Disorder subscale contains 13 items assessing the frequency of conditions such as trash, graffiti, drug dealers, and disorderly groups. The Victimization subscale, with 14 items, assesses frequency of worries about safety, such as walking alone, being robbed, or letting their child go outside alone. Response options for both subscales were revised to range from never (=1) to always (=5) (alphas = 0.91 and 0.92, respectively).

#### Body mass index

Trained research assistants measured height and weight using standard procedures [16,17]. Height and weight were measured in duplicate while the participant was wearing light indoor clothing and no shoes. Child BMI was calculated (weight(kg)/height(m)^2^) and used as a study outcome. Among adults, overweight was defined as having a BMI ≥ 25 and obese as having a BMI ≥ 30. Among children, BMI was converted to age- and gender-specific percentiles, and converted to z-scores using National Child Health Statistics growth charts for descriptive purposes [18]. In children, overweight is defined as having a BMI ≥ 85th percentile and obese as having a BMI ≥ 95th percentile.

#### Pubertal status

Pubertal status was included in analyses as a potential covariate because it has been associated with obesity in previous studies [19]. Pubertal status was assessed at all three assessments, via maternal report using the 5-item Pubertal Development Scale [20]. This measure, with versions for males and females, asks about physical development on characteristics associated with physical maturation, with response options ranging from *no* (1) to *yes, a lot* (3). Separate mean scores were calculated for each gender.

#### Physical activity monitoring

Actical accelerometer-based monitors (Philips Respironics, Bend, OR) were used to objectively measure physical activity. Actical contains an uniaxial accelerometer built from a cantilevered rectangular piezo-electric bimorph plate and seismic mass, which is sensitive to movement in all directions. The piezo-electric sensor is oriented in the monitor such that maximum sensitivity is obtained when the center of body mass is moved against gravity.

The monitors were affixed above the iliac crest of the right hip with an elastic belt and adjustable buckle. Children were instructed to wear the activity monitor continuously for three consecutive 24-h periods, including two weekdays and one weekend day (Wednesday through Saturday), and to remove the monitor only for bathing, showering or swimming. Sleep times, and times and reasons for monitor removal were recorded by the children and parents in an activity log.

At the completion of the data collection, the accelerometer data were downloaded into a computer. Data output included the time stamp and total accelerometer counts. In this study, 60 1-second values were summed together to generate one resultant raw activity datum for each 1-minute epoch. In the initial examination, data completeness was verified against the participant’s log. Times and reasons for removal of the monitor were coded in the file. Awake and sleep times were identified by visual inspection of a plot of activity counts per minute for each 24-h period. If continuous zeros for more than 20 minutes during awake periods were not accounted for in the participant’s log, it was assumed that the monitor was removed. A 24-hour day was required to have 1,000 minutes or more out of 1,440 minutes per day to be valid and useable. After this initial data treatment, activity counts were summed for each 24-hour period. Awake time was categorized into sedentary time, and light, moderate and vigorous levels of physical activity according to the thresholds derived using room respiration calorimetry [21]. Sedentary time (SED) was defined as activity energy expenditure (AEE) < 0.01 kcal^**.**^kg^-1**.**^min^−1^ or physical activity ratio (PAR) < 1.5, encompassing physical activities of minimal body movements in the sitting or reclined position. Light physical activity (LPA) was set at 0.01 < AEE < 0.04 kcal^**.**^kg^-1**.**^min^−1^ or 1.5 < PAR < 3.0, reflective of a low level of exertion in the standing position. Moderate physical activity (MPA) was set at 0.04 < AEE < 0.10 kcal^**.**^kg^-1**.**^min^−1^ or 3.0 < PAR < 6.0, and involved medium exertion in the standing position. Vigorous physical activity (VPA) level was set at AEE > 0.10 kcal^**.**^kg^-1**.**^min^−1^ or PAR > 6.0, reflective of activities at a high level of exertion in the standing position. In this analysis, SED was expressed as a percent of awake time (%SED) and MPA and VPA were combined and expressed as a percent of awake time (%MVPA).

### Statistical analysis

Statistical analyses were performed using SAS/STAT (version 13.1, SAS Institute, Inc., Cary, NC) and STATA (version 13.0, Statacorp, College Station, TX). Preliminary descriptive statistics and graphical presentation of the data over time were used to identify patterns in the data. To consider the effects of both mothers’ and fathers’ factors on their children’s BMI, %SED and %MPVA, all regression models were fit to data restricted to families with a biological mother and father or father figure in the child’s life.

#### Longitudinal regression models of factors influencing child BMI, %MVPA and %SED

Longitudinal regression models estimated the effects of parental, child and neighborhood factors on outcomes describing BMI, %MVPA and %SED at baseline, FU1, and FU2. For each model, baseline explanatory variables initially included child gender, child age, maternal age, paternal age, maternal education, paternal education, household income, maternal Spanish-language acculturation, paternal Spanish-language acculturation, number of household members, as well as maternal perceived neighborhood disorder and victimization. Additionally, time-varying explanatory variables included maternal BMI, paternal BMI, child BMI (in the %MVPA and %SED models) and mothers’ ratings of pubertal status. Initially, each time-varying explanatory variable was represented by two corresponding variables: (i) a variable holding baseline values, representing between person effects and (ii) a variable holding individual-level changes in values since baseline, representing within-person effects [22-24]. Each model was fit to pooled boys’ and girls’ data and two-way interactions between child gender and all other explanatory variables were initially included in the full model.

For each modeled outcome, a backward elimination procedure was implemented. In the first round of backward elimination, non-significant interactions (p > 0.05) and main effects (p > 0.20) involving baseline explanatory variables were removed from the model. Next, for each time-varying explanatory variable, the parameter estimates for (i) and (ii) were compared; if they significantly differed (p < 0.05), then both variables were retained in the model (unless subsequently removed by backward elimination); if the estimates for (i) and (ii) did not significantly differ, then (i) and (ii) were removed from the model and replaced with the original time-varying explanatory variable [22-24]. A final round of backward elimination removed any remaining non-significant time-varying explanatory variables (p > 0.20).

#### Cross-lagged panel models of child BMI, %MVPA and %SED

Cross-lagged models are widely used in the analysis of longitudinal data to provide evidence regarding the direction of causality between variables X and Y and to estimate the strength of the causal effects of each variable on the other. Cross-lagged models involve the estimation and comparison of correlation and regression coefficients between each variable measured at one wave and the other variable at the next wave.

**Figure 1:**
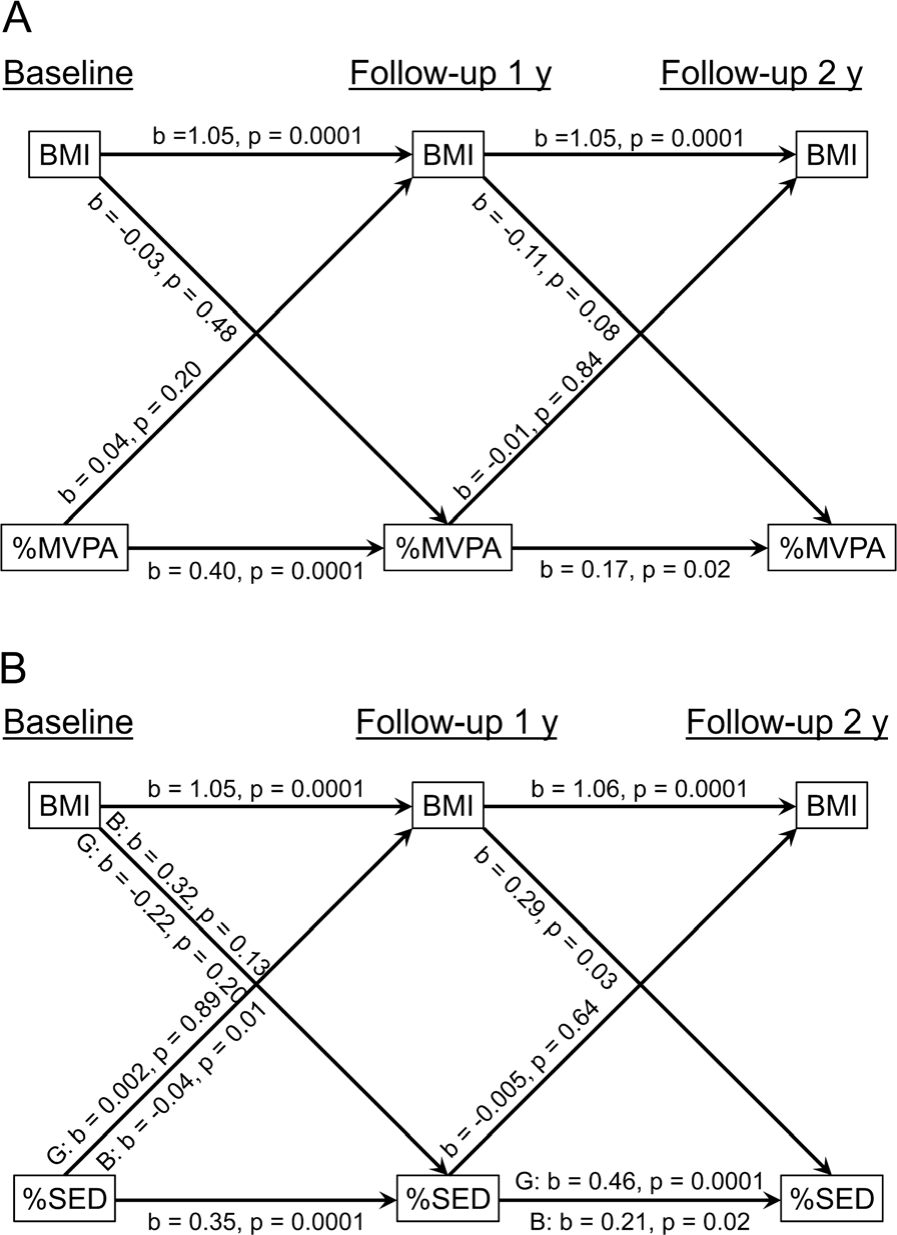
**Cross-lagged panel with BMI and %MVPA (A) and the cross-lagged panel with BMI and %SED (B) at follow-up 1 year and follow-up 2-years.** Where applicable, gender-specific effects are indicated for boys (B) and girls (G).

Cross-lagged panel models were fit via linear regression for two combinations of two outcomes, each assessed at baseline, FU1, and FU2: (A) child BMI and %MVPA and (B) child BMI and %SED. Each combination represents four equations, i.e., two outcomes that are each modeled at two occasions (FU1 and FU2; Figure 1). Each cross-lagged model included covariates describing child gender and child age at baseline, and the time-lagged pubertal status indicator (i.e., models of FU1 outcomes conditioned on baseline pubertal status; models of FU2 outcomes conditioned on FU1 pubertal status). All models were fit to data pooled across boys and girls and included all 2-way interaction terms involving the child gender indicator. A backward elimination process removed non-significant interaction terms (p > 0.05).

Each model was fit to 10 multiply imputed data sets created via a Markov Chain Monte Carlo method using SAS PROC MI. Imputation models were stratified by child gender. All parameter and standard error estimates were calculated by combining results across the imputed data sets [25].

## Results

The baseline sample included 282 Latino children (*n* = 133 males and *n* = 149 females), their 282 mothers, and 182 fathers or father figures (64%) who chose to participate. In the majority of households (93%), the biological father participated; in the remaining families, an adoptive father, stepfather or mother’s partner participated (7%). At baseline, mean (±SE) age of the children was 8.9 ± 0.05 years (Table 1). The majority of parents (74%) were from Mexico, and 95% of the children had been born in the U.S. Based on age- and gender-specific BMI percentiles, 52% of the children were classified as normal-weight, 19% as overweight and 29% as obese at baseline. Among these Latino families, most mothers (80%) and fathers (87%) were overweight or obese at baseline with mean BMIs of 30.1 ± 0.4 and 29.6 ± 0.4, respectively.

**Table 1:** **Characteristics of the Latino children over the two-year study period**

	**Baseline**	**Follow-up 1-year**	**Follow-up 2-years**
n	282	282	282
Gender	133 M/149 F	133 M/149 F	133 M/149 F
Puberty status	1.26 ± 0.02* M	1.32 ± 0.03 M	1.49 ± 0.03 M
	1.39 ± 0.03 F	1.57 ± 0.03 F	1.77 ± 0.03 F
Weight (kg)	38.1 ± 0.8	43.9 ± 0.9	49.0 ± 1.0
Height (m)	1.36 ± 0.005	1.43 ± 0.005	1.48 ± 0.006
BMI (kg/m^2^)	20.2 ± 0.3	21.2 ± 0.3	21.9 ± 0.3
BMI percentile	73.9 ± 1.6	73.5 ± 1.7	73.1 ± 1.8
BMI z-score	0.94 ± 0.06	0.92 ± 0.06	0.88 ± 0.07
Percentage normal-weight/overweight/obese (%)	52, 19, 29	50, 20, 30	50, 21, 29

*Abbreviations:* *F* female, *M* male, *BMI* body mass index. *Mean ± SE.

Among these Latino families, the average number of household occupants was 5.4 ± 0.1 with 2.7 ± 0.07 children. Mean maternal and paternal ages were 37.1 ± 0.4 years and 39.9 ± 0.5 years, respectively. Parents’ average years of education (fathers: 10.6 ± 0.2 years; mothers: 10.8 ± 0.2 years) was less than a high school education. Median family income was in the $40,000–50,000 range. Most parents were employed (72% of mothers, 88% of fathers). Employed parents’ occupational status ranged from unskilled worker (=1) to major professional (=9), with the average occupational status being semi-skilled worker for mothers (3.4) and skilled worker for fathers (3.7). Acculturation scores in Spanish language use (4.2 ± 0.07 mothers, 4.1 ± 0.08 fathers) were higher than acculturation scores in English language use (2.6 ± 0.08 mothers, 2.8 ± 0.08 fathers). Most interviews were conducted in Spanish (71% of mothers, 69% of fathers). Maternal and paternal perceptions of their neighborhoods were similar; the mean disorder (1.6 ± 0.05) and victimization (2.4 ± 0.08) scores were relatively low on a scale of 1 to 5.

Physical activity patterns of the Latino children were objectively assessed by accelerometry (Table 2). Total daily accelerometer counts (p = 0.04) and steps decreased linearly across the three study time points (p = 0.0001). Expressed in absolute minutes or as a percent of awake time, SED increased across time (p = 0.0001). In absolute minutes or as a percent of awake time, MVPA decreased across time (p = 0.02). At baseline, FU1 and FU2, the percent of children who accumulated ≥60 min/d MVPA was 46%, 36% and 34%, respectively.

**Table 2:** **Physical activity patterns of the Latino children measured by accelerometry over the two-year study period**

	**Baseline**	**Follow-up 1-year**	**Follow-up 2-years**
n	282	282	282
Total wear time (min/d)^c^	1,406 ± 2*	1,392 ± 3	1,374 ± 4
Awake time (min/d)^b^	834 ± 5	815 ± 5	811 ± 6
Total counts (counts/d)^a^	346,132 ± 15,762	302,866 ± 8,741	290,378 ± 9,447
Steps (steps/d)^c^	11,235 ± 305	10,201 ± 337	9,548 ± 354
Sedentary time (min/d)^c^	476 ± 7	493 ± 5	514 ± 5
Light physical activity (min/d)^c^	292 ± 5	267 ± 4	243 ± 4
Moderate physical activity (min/d)	63 ± 4	52 ± 2	50 ± 2
Vigorous physical activity (min/d)	2.1 ± 0.3	1.9 ± 0.2	2.2 ± 0.3
Moderate-vigorous physical activity (min/d)^a^	66 ± 4	55 ± 2	53 ± 2
Sedentary time (% awake time)^c^	57.4 ± 0.8	60.6 ± 0.6	63.6 ± 0.5
Light physical activity (% awake time)^c^	34.8 ± 0.5	32.7 ± 0.5	29.9 ± 0.4
Moderate physical activity (% awake time)	7.6 ± 0.4	6.4 ± 0.2	6.2 ± 0.2
Vigorous physical activity (% awake time)	0.26 ± 0.03	0.24 ± 0.02	0.28 ± 0.04
Moderate-vigorous physical activity (% awake time)^a^	7.9 ± 0.4	6.7 ± 0.2	6.5 ± 0.3

*Mean ± SE; Statistical notation: Linear time effect, adjusted for child gender and age at baseline: ^a^p-value < 0.05; ^b^p-value < 0.001; ^c^p-value < 0.0001.

In boys, there were significant positive correlations between BMI and %SED (r = 0.22 at baseline, 0.26 at FU1; p < 0.01) and negative correlations between BMI and %MVPA (r = −0.34 at baseline, −0.27 at FU1; p < 0.006). In girls, there were no significant correlations observed between BMI and %SED or %MVPA at baseline, FU1 and FU2.

### Longitudinal effects of parental, child and neighborhood characteristics on child BMI

Child BMI increased significantly across time (p = 0.0001) and a high degree of tracking or correlation was evident between successive observations (r_Spearman_ = 0.96, 0.97, p = 0.001). Regarding baseline covariates, a one-year difference in child age at baseline was associated with a time-averaged BMI difference of about +0.94 units (p = 0.0002; Table 3). Also, mothers with higher Spanish-language scores tended to have children with BMI values almost one-half unit higher (p = 0.04). Significant effects of time-varying explanatory variables included a change of 4.42 BMI units for every between-person, one-unit difference of the baseline pubertal status measure (p = 0.0001) and a change of 0.21 BMI units for every between-person, one-unit difference of baseline maternal BMI (p = 0.0001). Additionally, every between-person, one-unit difference on time-varying paternal BMI was associated with a contemporaneous difference of 0.11 child BMI units (p = 0.03). None of the modeled effects on child BMI were significantly modified by child gender.

**Table 3:** **Longitudinal regression models of parent, child and neighborhood factors on body mass index (BMI), moderate-vigorous physical activity (%MVPA) and sedentary time (%SED)**

	**BMI b, p-value**	**%MVPA b, p-value**	**%SED b, p-value**
**Time of assessment**			
FU1 v Baseline	0.89, p = 0.0001^a^	•	2.49, p = 0.0001^d^
FU2 v Baseline	1.63, p = 0.0001^a^	•	5.05, p = 0.0001^d^
**Baseline explanatory variables**			
*Child gender*			
boy vs. girl	0.79, p = 0.11	5.45, p = 0.0001^†^	−1.19, p = 0.08^§^
*Respondent age*			
Child	0.94, p = 0.002	−0.40, p = 0.03	2.48, p = 0.0001
Maternal	•	•	•
Paternal	•	−0.05, p = 0.02	0.06, p = 0.33^¥^
*Education level*			
Maternal	•	−0.10, p = 0.04	•
Paternal	•	•	•
*Spanish-language acculturation*			
Maternal	0.45, p = 0.04	•	−0.72, p = 0.14
Paternal	•	−0.125, p = 0.17	1.61, p = 0.01
*Household characteristic*			
Income	•	•	•
Size	−0.06, p = 0.07	•	−0.53, p = 0.005
*Neighborhood characteristic*			
Disorder	•	•	−1.03, p = 0.10
Victimization	•	•	•
**Time-varying explanatory variables**			
*Child BMI*			
BMI_tv_	n/a	−0.13^‡^, p = .0002	•
BMI_base_	n/a	•	•
BMI_Δ_	n/a	•	•
*Pubertal status (PS)*			
PS_tv_	n/e	−0.83, p = 0.06	•
PS_base_	4.42, p = 0.0001	•	•
PS_Δ_	•	•	•
*Maternal BMI*			
BMI_tv_	n/e	n/e	n/e
BMI_base_	0.21, p = 0.0001^b^	•	•
BMI_Δ_	0.05, p = 0.09^b^	−0.18, p = 0.004	0.51, p = 0.003
*Paternal BMI*			
BMI_tv_	0.11, p = 0.03	•	n/e
BMI_base_	•	•	0.15, p = 0.14^e^
BMI_Δ_	•	•	−0.74, p = 0.07^e^
**Interactions**			
*Gender-by-child BMI*	•		•
BMI_tv_ among boys		−0.22, p = 0.0002^c^	
BMI_tv_ among girls		−0.05, p = 0.19^c^	
*Gender-by paternal age*	•	•	
Paternal age among boys			−0.14, p = 0.10^f^
Paternal age among girls			0.26, p = 0.001^f^

*Abbreviations:* *BMI* body mass index, *FU1* Follow-up 1-year, *%MVPA* moderate-vigorous physical activity, *%SED* sedentary time, *FU2* Follow-up 2-years.

Statistical notation: interaction effects not tabled were removed via backward elimination; n/a: not included as an explanatory variable for the child BMI outcome; n/e: time-varying explanatory variable was never entered into the model because the corresponding between-person (_base_) and within-person (_Δ_) effects significantly differed; •: effect removed via backward elimination.

^a^omnibus 2 df p-value = 0.0001; ^b^p-value for difference between effect estimates = 0.0034.

^c^child gender-by-BMI interaction p-value = 0.02; ^d^omnibus 2 df p-value = 0.0001; ^e^p-value for difference between effect estimates = 0.04; ^f^child gender-by-paternal age interaction p-value = 0.0005.

^†^effect of child gender at the average child BMI value; ^‡^effect of time-varying child BMI averaged across boys and girls; ^§^effect of child gender at the average paternal age; ^¥^effect of paternal age averaged across boys and girls.

### Longitudinal effects of parental, child and neighborhood characteristics on child %MVPA

Conditional on modeled covariates, %MVPA levels did not significantly differ across assessments, but there was a moderate level of correlation across adjacent assessments (r_Spearman_ = 0.43, 0.39; p = 0.001). %MVPA was about 5.5% higher in boys than girls (p = 0.0001), but was lower as a function of child age at baseline (−0.4% per year of age, p = 0.03), paternal age at baseline (−0.05% per year of age, p = 0.002), and maternal education (−0.1% per year of education, p = 0.03). The effect of time-varying pubertal status was marginally significant: a one-unit (between person) increase on the pubertal status scale corresponded to an average contemporaneous reduction in %MVPA of about 0.83% (p = 0.06). A one-unit, within-person increase in maternal BMI since baseline corresponded to a reduction, since baseline, in %MVPA of about 0.18% (p = 0.004). Finally, the effect of time-varying child BMI was significantly stronger for boys than for girls (interaction p = 0.02): the effect of time-varying BMI was non-significant for girls, whereas it was significant and negative for boys: a one-unit (between-person) difference in boys’ BMI corresponded to about a 0.22% contemporaneous reduction %MVPA (p = 0.0002).

### Longitudinal effects of parental, child and neighborhood characteristics on child %SED

For %SED, time effects were significant: %SED increased an average of 2.5% per year (p = 0.0001). A moderate level of correlation across adjacent observations was also observed (r_Spearman_ = 0.38, 0.43; p = 0.001). Children who were older at baseline had a higher expected %SED values, about 2.5% higher for each year of child age (p = 0.0001). A one-unit increase in paternal Spanish-language score was associated with about a 1.6% increase in child %SED (p = 0.01), whereas a one-person increase in household size was associated with a 0.5% decrease in %SED (p = 0.005). A one-unit, within-person increase in maternal BMI since baseline corresponded to an increase, since baseline, in %SED of about 0.5% (p = 0.003). Finally, with respect to baseline explanatory variables, child gender modified the effect of paternal age on %SED (p = 0.0005); the effect for boys was non-significant whereas the effect of a one-year difference in paternal age tended to increase girls’ %SED by 0.3% (p = 0.001).

In all longitudinal regression models, neighborhood disorder and neighborhood victimization were not significantly predictive of child BMI, %MVPA or %SED.

### Cross-lagged panel including BMI and %MVPA

The cross-lagged panel with BMI and %MVPA demonstrated significant lagged relationships of BMI and %MVPA at FU1 and FU2, but no significant cross-lagged effects (Table 4; Figure 1A). While the lagged relationships for BMI were significant (p = 0.0001), the lagged relationships for %MVPA were weaker (p = 0.02–0.0001). There appeared to be diminution of effects across time: a one-percent between-person difference in %MVPA at baseline was expected to result in a 0.4% between-person difference in %MVPA at FU1, whereas the corresponding relationship was only 0.17 between FU1 and FU2. Age (p = 0.04) and BMI at baseline were significant predictors of BMI at FU1 (p = 0.0001). BMI at FU1 was a significant predictor of BMI at FU2 (p = 0.0001). Child gender (boys > girls) (p = 0.001) and prior %MVPA (p = 0.02–0.0001) were significant predictors of %MVPA at FU1 and FU2.

**Table 4:** **Cross-lagged panel models of child body mass index (BMI) and percent moderate-vigorous physical activity (%MVPA)**

**Dependent variable**	**Independent variable**	**Coefficient**	**T-value**	**P-value**
BMI at FU1	Gender	−0.335	−1.86	0.06
	Age	−0.211	−2.09	0.04
	Pubertal status	0.069	0.20	0.84
	BMI (at baseline)	1.054	53.5	0.0001
	%MVPA (at baseline)	0.035	1.28	0.20
BMI at FU2	Gender	−0.008	−0.04	0.97
	Age	−0.017	−0.13	0.89
	Pubertal status	−0.318	−1.02	0.31
	BMI (at FU1)	1.05	52.3	0.0001
	%MVPA (at FU1)	−0.006	−0.20	0.84
%MVPA at FU1	Gender	1.50	3.36	0.001
	Age	−0.465	−1.75	0.08
	Pubertal status	0.226	0.28	0.78
	BMI (at baseline)	−0.034	−0.71	0.48
	%MVPA (at baseline)	0.401	6.13	0.0001
%MVPA at FU2	Gender	1.97	3.51	0.0007
	Age	−0.105	−0.36	0.72
	Pubertal status	0.512	0.63	0.53
	BMI (at FU1)	−0.108	−1.81	0.08
	%MVPA (at FU1)	0.168	2.35	0.02

*Abbreviations*: *BMI* body mass index, *FU1* Follow-up 1-year, *%MVPA* moderate-vigorous physical activity, *FU2* Follow-up 2-years.

### Cross-lagged panel including BMI and %SED

The cross-lagged panel with BMI and %SED demonstrated lagged effects and also evidence for some cross-lagged effects (Table 5; Figure 1B). BMI had significant lagged effects from BL to FU2 (p = 0.0001). For %SED the lagged relationships were weaker than for BMI: a one-percent between-person difference in %SED at time *t* is expected to result in about a 0.35% between-person difference in %SED at time *t* + 1. However, the lagged effect of %SED at FU1 on %SED at FU2 showed gender-specific effects, with stronger effects among girls (0.46%, p = 0.0001) than boys (0.21%, p = 0.02). Significant cross-lagged effects included a positive effect of %SED at baseline on BMI at FU1 among boys only (b = −0.04%, p = 0.01) and a positive effect of BMI at FU1 on %SED at FU2 for both boys and girls.

**Table 5:** **Cross-lagged panel models of child body mass index (BMI) and sedentary time (%SED)**

**Dependent variable**	**Independent variable**	**Coefficient**	**T-value**	**P-value**
BMI at FU1	Gender	−0.352	−2.07	0.04
	Age	−0.197	−1.95	0.05
	Pubertal status	−0.029	−0.09	0.93
	BMI (at baseline)	1.055	53.7	0.0001
	%SED (at baseline)	−0.018	−1.66	0.10
	%SED (at baseline in girls)	0.002	0.14	0.89
	%SED (at baseline in boys)	−0.038	−2.56	0.01
BMI at FU2	Gender	−0.025	−0.13	0.89
	Age	0.004	0.03	0.97
	Pubertal status	−0.322	−1.04	0.30
	BMI (at FU1)	1.06	53.3	0.0001
	%SED (at FU1)	−0.005	−0.47	0.64
%SED at FU1	Gender	−10.6	−2.25	0.03
	Age	2.61	3.39	0.001
	Pubertal status	1.41	0.68	0.50
	BMI (at baseline)	0.050	0.35	0.73
	BMI (at baseline in girls)	−0.216	−1.31	0.20
	BMI (at baseline in boys)	0.316	1.55	0.13
	%SED (at baseline)	0.351	5.06	0.0001
%SED at FU2	Gender	−1.74	−1.53	0.13
	Age	0.821	1.18	0.24
	Pubertal status	−3.40	−1.69	0.10
	BMI (at FU1)	0.287	2.19	0.03
	%SED (at FU1)	0.334	5.32	0.0001
	%SED (at FU1 in girls)	0.458	5.07	0.0001
	%SED (at FU1 in boys)	0.209	2.40	0.02

*Abbreviations: BMI* body mass index, *FU1* Follow-up 1-year, *%SED* time, *FU2*, Follow-up 2-years.

## Discussion

This study sought to understand parental, child and neighborhood determinants and temporal patterns of BMI, %MVPA and %SED in Latino children as they approached adolescence. In this cohort, 48% of children were overweight or obese at 8–10 y and the vast majority of fathers and mothers were overweight or obese. Understanding the determinants of physical activity patterns is imperative in Latino children who are at high risk for later obesity and related cardiometabolic diseases.

First, we examined changes in BMI over the two-year period. As expected for normal growth, positive effects of child age and pubertal status at baseline on BMI were observed [18,26]. The BMI of these children increased about 0.95 units per year which maintained them along the 75th BMI percentile, on average. Even at this young age, a very high degree of tracking of BMI was observed over the two years [27,28]. Expected influences of paternal and maternal BMI on child BMI were demonstrated, representing genetic and environment influences [29,30]. Baseline maternal BMI, time-varying paternal BMI, and maternal Spanish language use were positively predictive of changes in child BMI. The high burden of parental obesity and the shared home environment pose a high risk for obesity among these Latino children.

Our observation that higher maternal Spanish-language scores were associated with higher child BMI is consistent with some [31-34], but not all reports [35,36]. Greater US acculturation is often equated to adopting a more Westernized diet and sedentary lifestyles, increasing the risk of obesity. However, the relationship is likely more complex. A systematic review examining the relationship between acculturation and diet in Latino adults living in the US indicated that less acculturation was related to more healthful diets [37], but not lower energy intake, fat intake or percent energy from fat. Parental Spanish acculturation was positively associated with child obesity in two studies [32,33]. In another study, Spanish language at home was associated with increased obesity risk in Central/South American but not Mexican origin children [31]. The effect of parental acculturation on the child most likely will depend on the acculturation measure used, length of stay in the US, age groups, and country of origin.

The major child determinants of %MVPA were age, gender, and BMI (in boys only). %MVPA was lower in older children and was significantly higher in boys than girls, with an adjusted mean difference of 5.5%. In boys, %MVPA was negatively associated with BMI. Of the parental factors, a change in maternal BMI, maternal education and paternal age were negatively associated with %MVPA. Interestingly, increases in maternal BMI predicted decreases in child %MVPA, again reflecting family lifestyles and shared environments.

The major child determinants of %SED were age and time of assessment with %SED increasing over the two-year period. Also, %SED increased with child age and tended to be higher among girls than boys. Of the parental factors, maternal BMI changes since baseline, paternal Spanish language use and paternal age (in girls only) were associated positively with %SED. Fathers who were older and more Spanish-acculturated may have practiced more traditional Latino behaviors, impeding their children’s participation in American sports and other physical activities. In British children, ages 8–10, higher paternal age was also associated with significant increases in sedentary behaviors, but no effect was seen on MVPA [38].

Timing of maturation affects both physical and psychobehavioral development, however, the effects on physical activity and inactivity have been equivocal [39]. In the Avon Longitudinal Study of Parents and Children (ALSPAC) study (n = 1351), biological maturity in boys only was inversely correlated with MVPA (r = −0.11; p = 0.01) and positively associated with sedentary activity (r = 0.10; p = 0.01) at 11 but not 13 y of age [39]. In our study, pubertal status was not significantly associated with %MVPA or %SED.

Moderate lagged associations of %MVPA (r_Spearman_ = 0.43, 0.39) and %SED (r_Spearman_ = 0.38, 0.43) were seen over the two-year period, somewhat stronger than other studies [40]. In Swedish school-aged children, low tracking in girls (*r* = 0.13–0.25) and low-moderate tracking in boys (*r* = 0.17–0.37) of MVPA and inactivity were observed over a two-year period [40]. Similar results were seen for physical activity and inactivity in middle school girls in the Trail of Activity in Adolescent Girls (TAAG) study; intraclass correlation coefficients ranged from 0.17 to 0.22 for self-report and 0.22 to 0.29 for 6-day accelerometry [41].

Our study design afforded us the opportunity to examine not only the lagged effects of child BMI, %MVPA and %SED, but also their cross-lagged effects on one another. Although there were no cross-lagged effects between BMI and %MVPA, the children with higher BMI at FU1 had larger increases in %SED at FU2. The heavier children may not have been inclined or encouraged to participate in sports activities, as they approached adolescence. Although BMI was positively correlated with %SED at baseline and FU1, boys with higher levels of %SED at baseline had smaller increases in BMI at FU1, conditioned on baseline BMI.

Of the environmental factors examined, only household size was shown to influence %SED. Neighborhood disorder and victimization were not significantly associated with child BMI, %MVPA or %SED. The parents’ perceptions of disorder and victimization for the San Francisco Bay Area were relatively low; apparently, any parental concerns did not restrain their children’s level of physical activity. Our results are consistent with a systematic review of 150 studies addressing environmental correlates of physical activity [42]. The only environmental correlates identified for children’s physical activity were father’s physical activity, child’s time spent outdoors, and school policies. Physical environment, socio-cultural environment, family structure, parental modeling, and parenting styles were unrelated to children’s physical activity. SES factors including family income, parental education and occupational status were not associated with children’s activity in the reviewed studies. Also, SES and parents’ perception of the neighborhood environment were unrelated to MVPA and sedentary time measured using accelerometry in two recent publications [43,44].

Most pediatric studies in the area of physical activity and health have been cross-sectional and therefore only established associations. In 897 Latino children aged 4–19 y, sedentary time was positively and strongly associated with adiposity (%fat mass) [10]. Adjusting for age, gender and %fat mass, sedentary time also was positively associated with fasting insulin and waist circumference. In 1862 British children aged 9–10 y, MVPA and VPA were inversely associated with adiposity indexes (BMI, fat mass and waist circumference) [45]. A few longitudinal studies have related physical activity to later health outcomes. A cross-sectional meta-analysis of 14 international studies found higher MVPA time in children and adolescents was associated with better cardiometabolic risk profiles (waist circumference, fasting insulin, triglycerides, HDL cholesterol, and systolic blood pressure) regardless of the amount of sedentary time [46]. In the longitudinal analysis, sedentary time and MVPA at baseline were not associated with follow-up waist circumference. However, higher waist circumference at baseline was associated with increased amounts of sedentary time at follow-up. In 280 British children aged 8–10 y, higher MVPA was predictive of lower 1-y changes in BMI [47].

In this study, lower %MVPA was seen in children with higher BMIs, even though cross-lagged effects of %MVPA on changes in BMI were not detected, possibly attributable to low dose of MVPA. At baseline, FU1 and FU2, only 46%, 36% and 34% of children accumulated the recommended ≥60 min/d MVPA, respectively. Conversely, higher BMI at the 1-y follow-up was shown to predict higher %SED at the 2nd year follow-up, perpetuating the strong tracking of BMI in these children. The beneficial effects of physical activity on child musculoskeletal health, cardiovascular fitness, and psychosocial well-being are indisputable, but the demonstrated effect sizes tend to be low to moderate [1]. Quantification of the long-term effects of physical activity and sedentary behaviors on health outcomes are hampered by our short-term measurement tools that capture only a snapshot of children’s activity.

## Conclusions

The major child determinants of physical activity (age, gender and BMI) and minor parental influences (maternal BMI and education, paternal age and acculturation) should be considered in designing interventions to promote MVPA and reduce sedentary time among Latino children as they approach adolescence. Parental influences on children’s physical activity through factors unique to each parent suggest that obesity prevention research should expand beyond parent–child dyads to include both mothers and fathers.

## Abbreviations

%MVPA: Percent moderate-vigorous physical activity
%SED: Percent sedentary physical activity
BMI: Body mass index
FU: Follow-up
AEE: Activity energy expenditure
PAR: Physical activity ratio
LPA: Light physical activity
MPA: Moderate physical activity
VPA: Vigorous physical activity
